# Effects of radioiodine administration on serum concentrations of matrix metalloproteinases, adiponectin and thrombospondin

**DOI:** 10.1186/1756-6614-6-S2-A40

**Published:** 2013-04-05

**Authors:** Andrzej Lewiński, Anna Brona, Diana Jędrzejuk, Anna Bohdanowicz-Pawlak, Krzysztof C Lewandowski, Małgorzata Bieńkiewicz, Andrzej Milewicz

**Affiliations:** 1 Department of Endocrinology and Metabolic Diseases, Medical University of Lodz, Poland; 2 Department of Endocrinology, Diabetology and Isotope Therapy, Medical University of Wroclaw, Poland; 3 Department of Quality Control and Radiation Protection, Medical University of Lodz, Poland

## Introduction

In order to assess long-term safety of radioactive iodine administration in treatment of thyrotoxicosis, we assessed concentrations of selected markers of risk cardiovascular disease, i.e. matrix metalloproteinase 2 (MMP-2), its main inhibitor TIMP-2, matrix metalloproteinase 9 (MMP-9), its main inhibitor TIMP-1, as well concentrations of anti-inflammatory adiponectin and pro-inflammatory thrombospondin.

## Material and methods

The study involved 23 patients (3 males) age 53±12 (mean±SD) years treated with radioiodine for thyrotoxicosis. Serum concentrations TSH, fT4, fT3, MMP-2, MMP-9, TIMP-1, TIMP-2, adiponectin and thrombospondin were measured just before radioiodine administration (visit 1), and subsequently, after 7 days (visit 2), 3 months (visit 3), six to eight months (visit 4) and 15-18 months after radioiodine administration (visit 5).

## Results

There were no acute changes in serum concentrations of MMP-2, MMP-9, TIMP-1 and TIMP-2 adiponectin and thrombospondin (visit 1 versus visit 2). Subsequently, however, there was no change in serum MMP-9 or thrombospondin, but an increase in MMP-2 (from 393±106 ng/ml, to 774 ± 424 ng/ml), TIMP-1 (from 177 ± 76 ng/ml to 296 ± 118 ng/ml), TIMP-2 (from 136±44 ng/ml to 168±41 ng/ml), and adiponectin (from 16442±9490 ng/ml to 23518±9840 ng/ml), visit 1 to visit 5 respectively, p<0.01). Further analysis, however, revealed no significant change in MMP-2/TIMP-2 ratio, but there was a significant decrease in MMP-9/TIMP-1 ratio (p<0.05), suggestive of possible decrease in concentrations of free MMP-9.

## Conclusions

Our data reveal a significant and sustained increase in serum adiponectin as well as possible decrease of concentration of free MMP-9 after radioiodine administration. This might indicate overall safety of radioiodine treatment of thyrotoxicosis in terms of the risks of cardiovascular disease.

